# Predicting wheat yield and grain quality with UAV multispectral imagery and deep learning

**DOI:** 10.3389/fpls.2026.1812052

**Published:** 2026-05-28

**Authors:** Mohammad Maruf Billah, Maitiniyazi Maimaitijiang, Swas Kaushal, Bruce Millett, Shahid Nawaz Khan, Jyotirmoy Halder, Jonathan Kleinjan, Sunish K. Sehgal

**Affiliations:** 1 Department of Geography and Geospatial Sciences, Geospatial Sciences Center of Excellence, South Dakota State University, Brookings, SD, United States; 2 Department of Agronomy, Horticulture, and Plant Science, South Dakota State University, Brookings, SD, United States

**Keywords:** deep learning (DL), high-throughput phenotyping (HTP), protein and test weight estimation, unmanned aerial vehicles (UAV), wheat yield

## Abstract

Bread wheat (*Triticum aestivum* L.) is a major staple crop, and timely, in-season prediction of grain yield (GY) and grain quality traits, grain protein content (GP), and grain test weight (TW), is critical for informed management and field-based high-throughput phenotyping (HTP). Unmanned Aerial Vehicle (UAV) remote sensing, coupled with artificial intelligence and deep learning (DL), offers a practical pathway for rapid, plot-scale trait estimation. Here, we investigate the value of multitemporal, multispectral UAV imagery for predicting winter wheat GY, GP, and TW, and we systematically compare two modeling paradigms: (1) handcrafted feature-based workflows that use plot-aggregated spectral and texture descriptors derived from UAV imagery, and (2) image-based, end-to-end workflows that learn directly from plot-level reflectance image chips. During the 2022 growing season, multispectral UAV data were collected repeatedly over seven experimental wheat sites in South Dakota, USA. For handcrafted feature-based modeling, we evaluated Support Vector Regression (SVR) and Random Forest Regression (RFR), along with DL models including a feedforward Deep Neural Network (DNN) and a one-dimensional Convolutional Neural Network (1D-CNN). For end-to-end image-based modeling, we implemented 2D-CNN, 3D-CNN, and a hybrid 2D-CNN–LSTM architecture to leverage both spatial information and multi-date dependencies. Our results show that: 1) the image-based modeling workflow yielded comparable to slightly better performance than the handcrafted feature-based modeling workflow across wheat GY, GP, and TW predictions; 2) 3D-CNN outperformed all other methods with R^2^ of 0.65, 0.61 and 0.69 for GY, GP and TW estimations, respectively; 3) multitemporal UAV data outperformed the data collected from a single growth stage; and UAV data from wheat Feekes 10 (booting) stage yielded slightly better estimation results compared to the data collected from other growing stages, with R^2^ of 0.62, 0.55, and 0.62 for GY, GP, and TW estimations, respectively. The results indicate that DL applied to high-resolution multitemporal and multispectral UAV imagery holds strong promise for predicting winter wheat yield and grain quality during the growing season, while also informing HTP efforts and site-specific management.

## 1 Introduction

Wheat is the most extensively grown cereal across the globe and is a major food source for about 35% of the world’s population and contributes essential protein and daily caloric intake (Kaplan Evlice et al., 2024). The growing pressures of population expansion, climate change, and limited land and water resources pose significant threats to global food security (Ghosh et al., 2024). Therefore, innovative strategies and technologies are essential to improving wheat production and quality. Recent advancements in wheat breeding, high-throughput phenotyping (HTP), and precision farming have emerged as key solutions and technologies for enhancing wheat grain yield and quality. Within the framework of HTP and precision farming, timely and rapid monitoring, and in-season prediction of wheat grain yield (GY) and quality traits, such as grain protein (GP) content and test weight (TW), play a pivotal role. HTP technologies facilitate rapid, large-scale assessment of crop traits, enabling breeders to efficiently identify and select superior wheat varieties (Fei et al., 2022). Meanwhile, precision farming integrates timely wheat growth and health status information to optimize in-season management decisions such as irrigation, fertilization, and disease control, while also informing broader business and marketing strategies (Meroni et al., 2021).

The traditional approach to wheat growth monitoring, phenotyping, and field management often relies on in-person field visits, visual inspections, and manual data collection, which is time-intensive, labor-demanding, and expensive. These limitations hinder its efficiency, particularly in large-scale wheat breeding programs and precision farming applications (Li et al., 2021). Remote sensing enables efficient and economical earth observation and crop monitoring, supporting diverse agricultural applications. Notably, recent progress in Unmanned Aerial Vehicle (UAV)-based remote sensing enables flexible data acquisition and the collection of high-resolution, multi-sensor imagery, thereby enhancing fine-scale crop monitoring and modeling, as well as field-based fine-scale HTP (Khuimphukhieo and da Silva, 2025).

By leveraging conventional machine learning (ML), as well as advanced deep learning (DL) models, high-resolution monitoring of crop conditions and the prediction of yield- and quality-related parameters have become increasingly feasible (Liu et al., 2025). In these applications, canopy spectral descriptors, such as vegetation indices computed from UAV RGB, multispectral, or hyperspectral imagery, are most commonly used as input features for modeling diverse plant traits. These include biophysical parameters such as crop leaf area index, and above-ground biomass, biochemical traits such as leaf nitrogen content, chlorophyll content, water content, and pigment levels, as well as physiological indicators such as stomatal conductance (Gano et al., 2024). Furthermore, UAV-based spectral information has also been exploited to estimate crop grain yield and quality traits such as protein and oil concentration (Dilmurat et al., 2022).

In addition to commonly used spectral features derived from UAV imagery, texture features obtained from Gray Level Co-occurrence Matrix (GLCM) calculations, as well as combinations of spectral and texture features, have been widely employed for various crop trait estimations and yield predictions. Specifically for wheat, spectral and textural features, or their combinations, derived from UAV-acquired multispectral and hyperspectral imagery have been used to predict crop final grain yield production and grain protein concentration (Dilmurat et al., 2022; Kang et al., 2024). However, wheat TW, which refers to the weight of wheat that fills a standard volume, is an important indicator of grain quality, and has been less explored in UAV-based remote sensing-based prediction approaches (Kaushal et al., 2024). While UAV remote sensing studies in wheat have increasingly focused on GY and GP, relatively few studies have specifically investigated the in-season prediction of TW using UAV multispectral imagery. For example, Kaushal et al. (2024) evaluated UAV-based phenotyping to improve TW prediction in winter wheat breeding, and Vatter et al. (2022) used UAV-based multispectral imaging for preharvest prediction of durum wheat grain yield and quality traits, including TW. Other TW-related studies are mainly based on postharvest or grain-level measurements, such as small-volume specific gravity or grain-based spectral measurements, rather than field-scale UAV phenotyping (Donelson et al., 2002; Assadzadeh et al., 2022). Since TW is an important grain-quality trait in wheat grading and market assessment (Danciu, 2022), evaluating its predictability as a primary grain-quality trait alongside GY and GP can address an underexplored area in UAV-based wheat trait prediction.

In UAV remote sensing studies focused on plant trait estimation and yield forecasting, especially in HTP, researchers typically rely on spectral, textural, and other feature sets extracted from UAV imagery. These features are typically averaged at the research or breeding-plot level and correlated with corresponding plot-level grain yield or other plant traits (hereafter denoted as the feature-based method or workflow) (Tanaka et al., 2024). Feature extraction and engineering are often employed to generate informative handcrafted (i.e., manually extracted) features, which are then used as input variables in ML models (Tanaka et al., 2024). To streamline this process, several tools and software packages have been developed to automate the extraction of various feature types, especially for HTP use cases. Notable examples include the FIELDimageR package (Matias et al., 2020) built on OpenDroneMap, and the Orfeo Toolbox (OTB) (Grizonnet et al., 2017), as well as Hyperfidelis software toolkit (Sagan et al., 2024), etc. These tools have been widely adopted in UAV image processing pipelines to support model development for plant trait estimation and yield prediction. However, handcrafted feature-based approaches typically require separate steps for feature extraction and selection, making them time-consuming, computationally inefficient, and highly dependent on user expertise (Hartling et al., 2019; Sagan et al., 2021). Additionally, handcrafted features such as vegetation indices, texture, shape, and geometrical attributes may not effectively capture the complex patterns associated with plant traits and can potentially lose important spatial and contextual information present in raw images when averaged at the plot level (Sagan et al., 2021; Shoaib et al., 2023).

Alternatively, instead of relying on image-derived features and extensive preprocessing steps, raw UAV images can be directly used as input to deep learning models (referred to in this work as the end-to-end direct image-based method or workflow) for plant traits estimation (Bhadra et al., 2024). The direct image-based workflow processes UAV imagery in an end-to-end manner, eliminating the necessity for feature engineering and plot-level averaging. By operating directly on the imagery, this approach retains rich spatial structure, spectral signatures, and textural cues, allowing models to learn intricate spatial patterns and contextual dependencies that are frequently discarded by handcrafted, feature-based pipelines. Consequently, it can often improve the estimation of complex plant traits such as biomass, grain yield, and protein concentration (Bhadra et al., 2024; Meraj et al., 2024). Additionally, by eliminating time-intensive preprocessing and manual feature engineering, these approaches improve scalability and streamline the analysis pipeline, making them well-suited for large-scale agricultural applications (Parida et al., 2024; Sagan et al., 2021).

In terms of predictive modeling algorithms in the above-mentioned feature-based workflow, one-dimensional handcrafted features such as vegetation indices or textural metrics are averaged at the plot level and serve as input variables for ML models. This workflow commonly employs traditional ML techniques, including Random Forest (RF), Gradient Boosting Machine (GBM), and Support Vector Machine (SVM), as well as DL structures such as Deep Neural Networks (DNN) and one-dimensional Convolutional Neural Networks (1D-CNN), which are well-suited for handling 1D feature inputs (dos Santos Felipetto et al., 2025; Kang et al., 2024; Zhang et al., 2021). While these models have shown promise, their reliance on simplified feature representations may hinder their capacity to capture the complex spatial, spectral, and temporal patterns inherent in high-resolution UAV imagery (Sagan et al., 2021). In terms of the direct image-based workflow, DL approaches such as two-dimensional and three-dimensional Convolutional Neural Networks (2D-CNN and 3D-CNN), hybrid 2D-CNN–Long Short-Term Memory (LSTM) networks (denoted as 2DCNN-LSTM), as well as emerging architectures like Vision Transformers (ViTs), can be employed, as these architectures can use preprocessed UAV imagery (i.e., radiometrically and geometrically corrected image patches) directly as input, without relying on handcrafted spectral or texture features (Sagan et al., 2021; Shoaib et al., 2023; Yang et al., 2023). By leveraging appropriate DL models, the direct image-based workflow can automatically learn hierarchical and multi-scale features from multitemporal, multispectral UAV imagery, and capture spatial structures, spectral variations, and temporal dynamics critical for modeling complex agronomic traits, and potentially deliver improved prediction performance (Bhadra et al., 2024; Yang et al., 2023). However, the direct image-based workflow remains largely underexplored for crop grain yield and quality prediction, particularly with respect to comparative analysis of feature-based and imagery-based workflows in the context of field-based wheat HTP programs.

In addition to the predictive modeling workflows and algorithms used, the timing of UAV image acquisition is also an important factor affecting the accuracy of in-season prediction of wheat traits. Although multitemporal UAV observations often improve prediction performance by capturing crop development across phenological stages (Camenzind and Yu, 2024), repeated flights increase cost, labor requirements, and operational complexity and may not always be feasible in field-based HTP or precision agriculture applications due to weather constraints such as rain and high winds. Therefore, identifying the optimal single or key growth stages for predicting specific wheat traits is important for developing prediction systems that are accurate and efficient. Previous studies have reported inconsistent findings regarding the most informative growth stage for wheat traits prediction. Walsh et al. (2022) found that imagery acquired at Feekes 5 was more useful than Feekes 10 for grain-yield prediction, whereas neither stage provided strong prediction for grain protein. Zhou et al. (2021) reported the highest yield prediction accuracy at the heading stage and further showed that combining data from multiple stages improved performance compared with single-stage inputs. Additionally, Heinemann et al. (2025) found that later growth stages, particularly around grain filling, were more effective for grain-yield prediction. These contrasting findings suggest that the optimal imaging time is not consistent across studies and likely depends on the target trait, crop developmental stage, sensor-derived information, and modeling approach. As a result, it remains unclear which single stage or key growth stages are most informative for jointly predicting GY, GP, and TW based on UAV observations. Thus, a systematic evaluation and identification of the optimal imaging time from multi-stage UAV observations is needed for accurate and efficient wheat GY, GP, and TW predictions.

Therefore, this work aims to (1) investigate the potential of feature-based and imagery-based workflows for predicting GY and quality traits GP and TW in field-based wheat HTP using UAV multitemporal multispectral imagery; (2) evaluate the performance of traditional ML methods such as Random Forest (RF) and Support Vector Machine (SVM), alongside DL methods including DNN, 1D-CNN, 2D-CNN, 3D-CNN, and 2DCNN–LSTM; and (3) identify the optimal UAV imaging time for accurate prediction of GY, GP, and TW in South Dakota, US.

## 2 Test sites and data

### 2.1 Test sites and experimental setup

**Figure 1:**
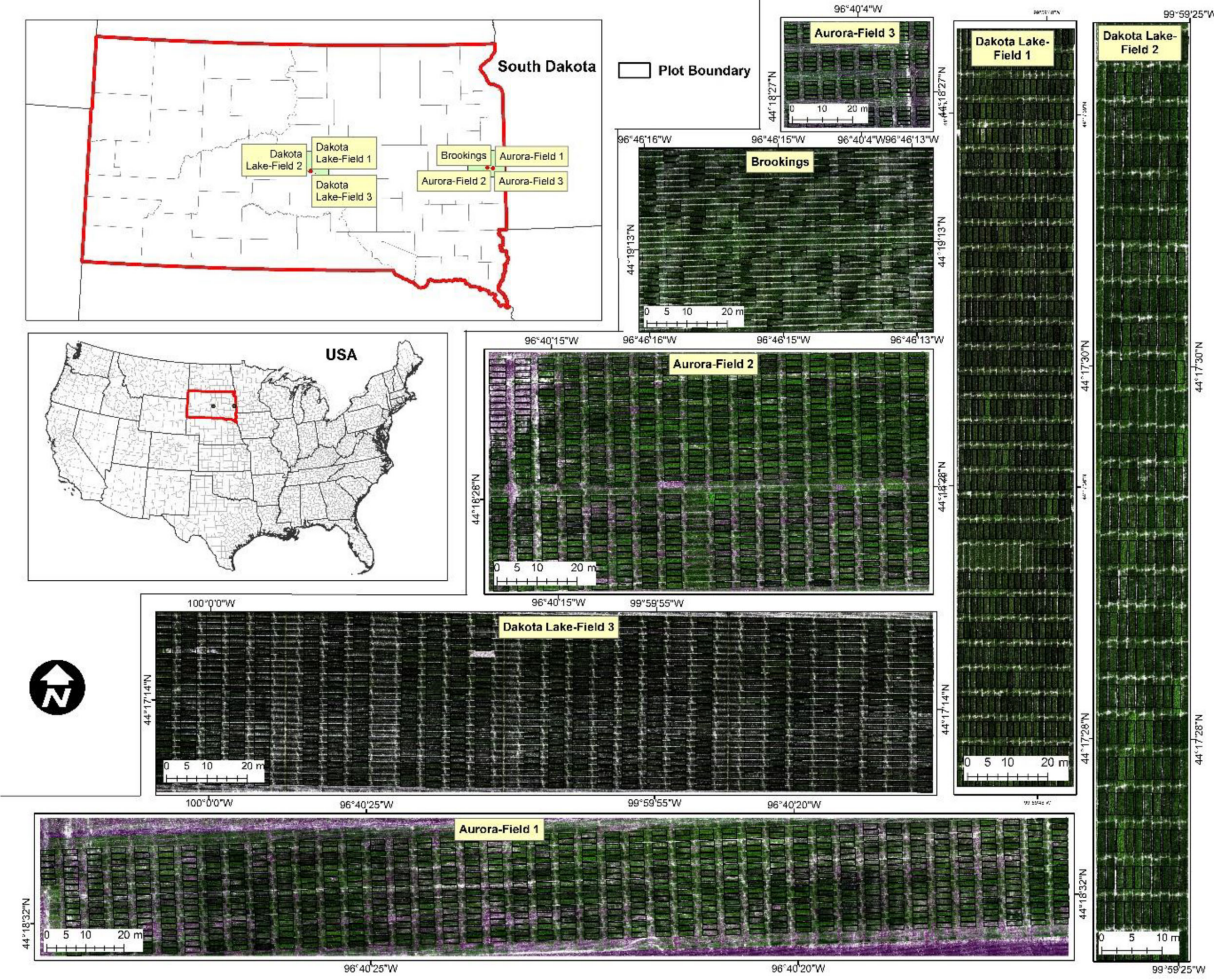
Geographical locations of the 7 winter wheat experimental fields established in Central and Eastern South Dakota, USA. and the corresponding field layout for each site. Panels on the right and bottom display example UAV-derived orthomosaics and plot boundaries for the Brookings, Aurora (Fields 1–3), and Dakota Lake (Fields 1–3) sites.

Seven experimental winter wheat fields in eastern and central South Dakota (SD) (**Figure 1**) were used in this research. These seven were established for wheat breeding research for the 2021–2022 growing season. Four fields are located in Brookings County (eastern SD), and three are in Hughes County (central SD). These sites experience diverse climatic and environmental conditions. The four eastern fields have a partly cloudy climate year-round, with warm summers, snowy and windy winters, and average temperatures ranging from –13 °C to 28.33 °C, with 559 mm of precipitation annually. The three central fields record the highest average temperature (31.27 °C) in July and the lowest (–13.72 °C) in January, with an annual precipitation of 482.6 mm. Regional variability in soil conditions is also evident. The four eastern fields contain moderately well-drained soils formed from loess over glacial till, predominantly located on foot slopes and within swales. The topography in the three central fields ranges from undulating to sloping, with well-drained soils consisting of clay loams, loams, and silt loams.

A total of approximately 3,339 research plots, each with plot dimensions of 1.53 m × 3.96 m, were established across the seven fields. These trials were planted in September 2021 with multiple winter wheat varieties using a randomized complete block design with three replications, meaning that each genotype was repeated three times within each field to reduce the effect of local field variability. Plots were established under a no-till management system, in which seeds were planted directly into untilled soil and crop residue from the previous season was retained on the soil surface, in seven rows spaced 20 cm apart, with a seeding rate of 300 seeds m^−2^. All sites relied exclusively on natural rainfall (rainfed conditions) and followed locally recommended management practices to ensure optimal crop growth and grain yield.

### 2.2 Data collection

#### 2.2.1 Field data acquisition

**Figure 2:**
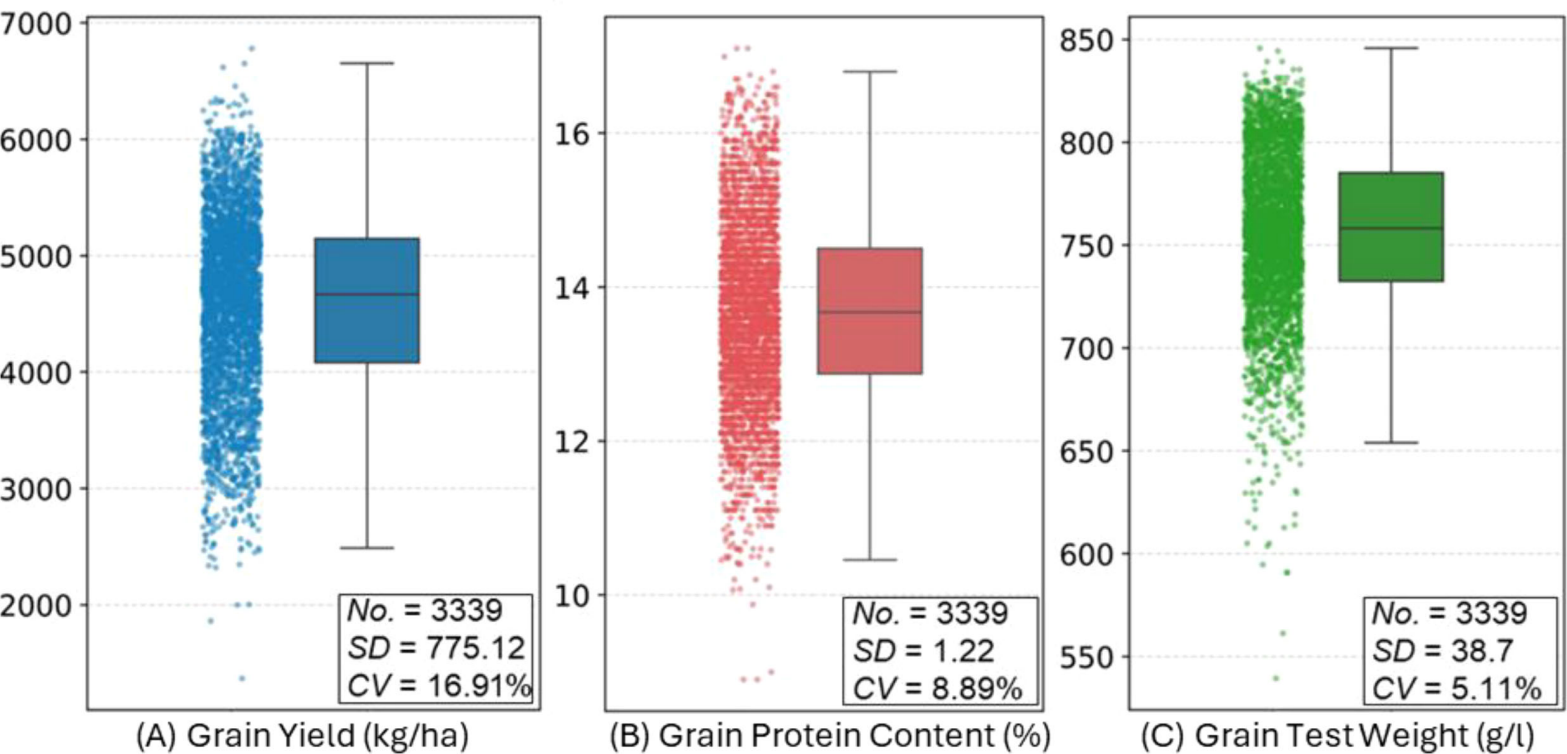
Distributions of grain yield (kg/ha) **(A)**, grain protein content (%) **(B)**, and grain test weight (g/l) **(C)** for all observations (n = 3339). Dots represent individual data points, while boxplots show the median, interquartile range, and 1.5×IQR whiskers. Each inset summarizes the sample count (No.), standard deviation (SD), and coefficient of variation (CV) for the corresponding trait.

Wheat was harvested at physiological maturity in July 2022 using a small-plot combine (Zurn, Westernhausen, Germany), and GY was recorded for each research plot. GP and TW were subsequently determined in the laboratory using an Infratec™ 1241 Grain Analyzer (FOSS North America, Eden Prairie, MN, USA). To standardize measurements and enable direct comparisons across variable grain moisture conditions, both GY and GP were adjusted to a 13% moisture basis. GY, GP, and TW were expressed in kg/ha, percentage (%), and g/l, respectively. Distribution and summary statistics of ground truth wheat grain yield and quality parameters are presented in **Figure 2**.

#### 2.2.2 UAV image acquisition

**Figure 3:**
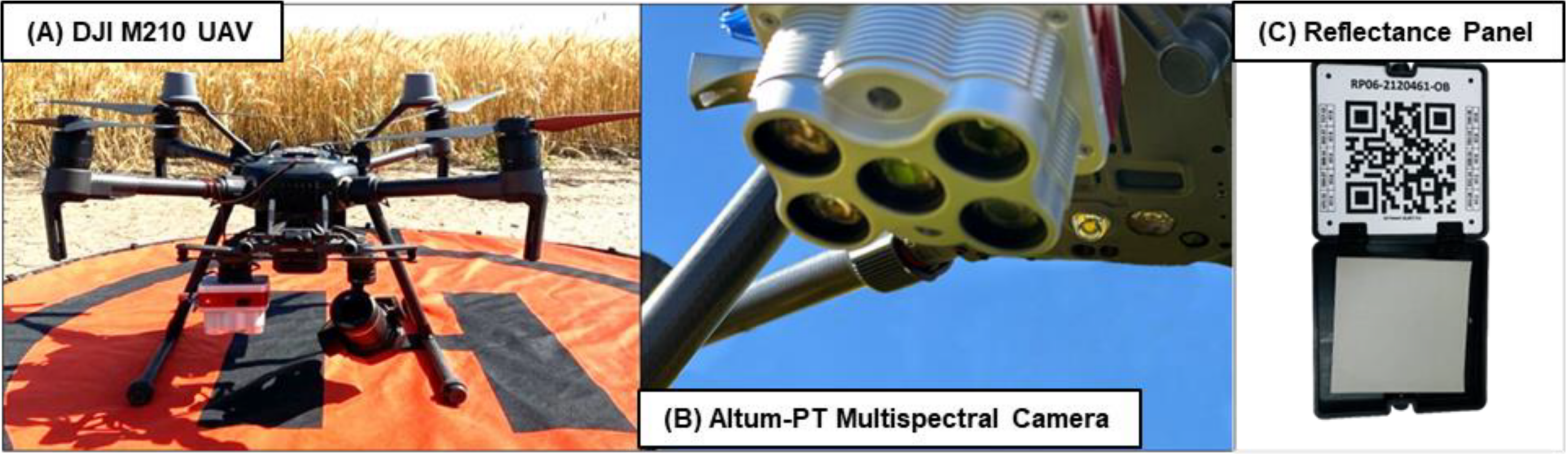
UAV-based data acquisition system was used in this study. **(A)** DJI Matrice 210 UAV platform; **(B)** multispectral sensor mounted on the UAV; and **(C)** calibrated reflectance panel used for radiometric calibration of imagery.

Between May and July 2022, UAV data were collected on five separate dates at each experimental site (May 18, June 3, June 9, June 27, and July 7), aligning with the wheat growth stages of Jointing (Feekes 6), Flag Leaf (Feekes 8), Booting (Feekes 10), Heading (Feekes 10.5), and Milky Ripe (Feekes 11), respectively. UAV imagery was collected with a DJI Matrice 210 RTK platform (**Figure 3A**) equipped with a MicaSense Altum-PT multispectral sensor (3.2 MP; 2064 × 1544 pixels) (**Figure 3B**). The sensor captures five discrete wavebands: blue (475 nm), green (560 nm), red (668 nm), red-edge (717 nm), and near-infrared (842 nm). Prior to each flight mission, the MicaSense calibrated reflectance panel (model RP06-2120461-OB) (**Figure 3C**) was imaged to enable radiometric calibration. The reflectance panel served as a reference target with known band-specific reflectance values. In addition, an onboard Downwelling Light Sensor (DLS) mounted on the top of the UAV was also used to record downwelling illumination conditions and compensate for potential changes in lighting conditions during flight (Kaushal et al., 2024). The flight mission for each field was conducted at an altitude of 45 m with 75% frontal and side overlaps. To maintain consistent acquisition conditions, the UAV operated at a fixed ground speed of 4 m/s, with flights conducted during the day from 10:00 a.m. to 2:00 p.m. under clear-sky, low-wind, and visually stable illumination conditions.

#### 2.2.3 UAV data processing

Multispectral imagery from each UAV mission was processed in Pix4Dmapper (Pix4D SA, Lausanne, Switzerland) to generate radiometrically calibrated orthorectified mosaic images. Radiometric calibration was applied within the Pix4D workflow using pre-flight reflectance panel image captures together with irradiance measurements provided by the onboard DLS (Kaushal et al., 2024). The reflectance panel provided band-specific reference reflectance values, and the DLS was used to account for variations in incoming light during image acquisition (Janjua et al., 2026). The combined use of reflectance panel imagery and DLS measurements helped reduce radiometric inconsistency caused by changing ambient light conditions and improved comparability among image acquisitions across dates. Additional processing, including layer stacking, image co-registration, and the generation of plot boundary polygons, was conducted in ArcGIS Pro software (Esri, Redlands, CA, USA).

## 3 Methodology

### 3.1 Feature extraction

Spectral metrics and texture descriptors were computed at the plot scale from the UAV imagery and used as model inputs for feature-based ML models in the estimation of wheat GY, GP, and TW. Specifically, spectral features such as the original five reflectance bands from the multispectral imagery, as well as a range of commonly used vegetation indices, were calculated and averaged at the plot level. Following Maimaitijiang et al. (2025), we constructed 48 spectral predictors/features from the UAV multispectral data, including the five bands reflectances and derived vegetation indices responsive to canopy nutrient content, biomass accumulation, and health-related variability. All spectral variables were aggregated to the plot scale using mean statistics.

Beyond the spectral variables, we also derived plot-scale texture descriptors from the UAV multispectral images using the gray-level co-occurrence matrix (GLCM) framework (Haralick et al., 1973). GLCM summarizes local spatial structure by quantifying how frequently different pixel intensity levels occur in specified spatial relationships, providing a statistical description of image texture. These texture measures complement spectral variables by capturing canopy structural variability and within-plot heterogeneity that may relate to differences in growth, vigor, and plant condition. Following Dhakal et al. (2023), we computed 40 GLCM texture features in total, consisting of eight texture metrics for each of the five multispectral bands. The combined spectral and texture feature set was subsequently employed as input to ML and DL models for predicting wheat grain yield and quality parameters.

### 3.2 Modeling methods

In this research, two distinct modeling frameworks were implemented for the prediction of wheat GY, GP, and TW. The first method utilized a suite of handcrafted parameters, specifically spectral and textural features extracted from plot-scale image segments, as primary input variables. The second approach leveraged raw image data as direct inputs for machine learning architectures to automate trait estimation without manual feature engineering.

#### 3.2.1 Handcrafted feature-based modeling approach

Handcrafted image features (i.e., spectral indices and texture metrics) were assembled as predictors for conventional ML models, including SVR and RFR, as well as DL models, such as DNN and 1D-CNN, to predict wheat GY, GP, and TW. The conventional ML methods and the DL architectures considered in this study operate on one-dimensional predictor vectors. Accordingly, we derived 48 spectral indices and 40 texture descriptors per plot from each flight/day’s UAV imagery. Considering five flights, this resulted in a total of 440 features per data point (or plot), calculated as 5 flights × (48 spectral features + 40 texture features) = 440 features.

##### 3.2.1.1 Support vector regression

SVR is the regression counterpart of the Support Vector Machine (SVM) framework, developed to predict continuous-valued outcomes (Cortesc, 1995). It maps input features, such as spectral and texture descriptors, into a higher-dimensional space via a kernel function to capture complex, potentially nonlinear relationships. SVR identifies an optimal hyperplane that maximizes the decision margin within a defined epsilon-tolerance, effectively balancing the minimization of training error with the maintenance of model generalization. SVR has been widely reported in the literature for monitoring and modeling crop traits using a range of remote sensing platforms. In many studies, SVR was adopted as a baseline approach to provide a performance benchmark, enabling researchers to quantify the improvements offered by more advanced deep learning approaches (Kok et al., 2021). For the SVR-based analysis, the input was organized into a 1D array comprising 440 distinct features per sample. The corresponding input feature dimension is expressed as (440), representing a single 1D array of 440 features per sample.

##### 3.2.1.2 Random forest regression

RFR operates as an ensemble framework that aggregates the predictions of numerous individual decision trees generated during training phase to enhance predictive precision and mitigate the risk of overfitting (Breiman, 1996). By incorporating randomness through the subsampling of both data instances and variables during the tree-building process, RFR minimizes model variance and enhances predictive robustness. Its inherent capacity to process high-dimensional inputs and capture intricate, nonlinear relationships makes it a premier choice for remote sensing applications, particularly in the context of plant traits estimation and yield forecasting. Consequently, it remains a standard reference model used to contextualize the advancements and accuracy gains of emerging deep-learning frameworks (Pokhariyal et al., 2023). Similar to SVR, the input of RFR was organized into a 1D array comprising 440 distinct features per sample.

##### 3.2.1.3 Deep neural networks

**Figure 4:**
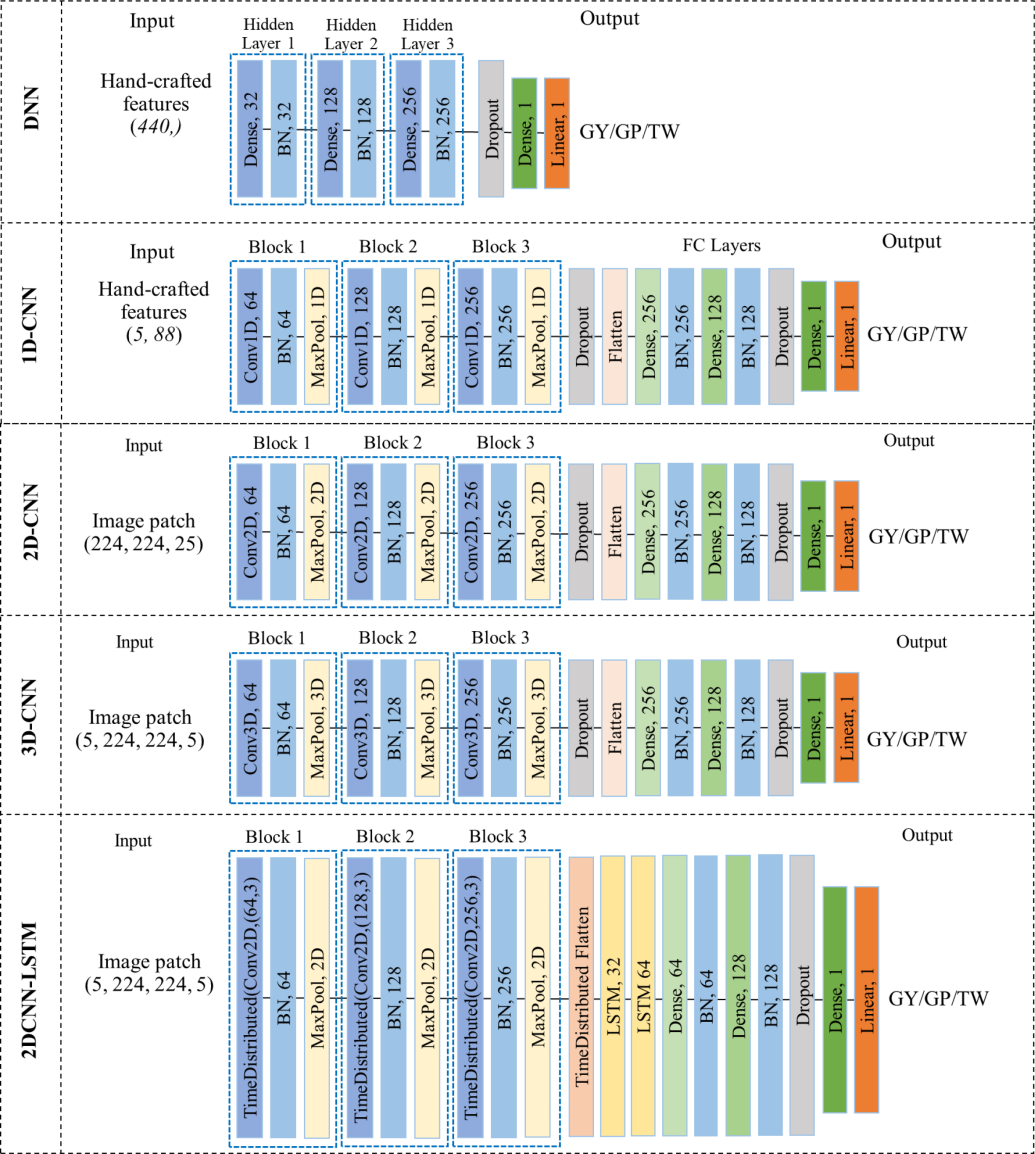
Input configuration, model architecture, and outputs of the deep learning models used in this study. From top to bottom, the rows show: a deep neural network (DNN) and a one-dimensional convolutional neural network (1D-CNN), which were designed to process UAV-derived handcrafted features for wheat trait prediction; and a two-dimensional convolutional neural network (2D-CNN), a three-dimensional convolutional neural network (3D-CNN), and a two-dimensional convolutional neural network combined with long short-term memory (2D-CNN–LSTM), which were designed to directly process UAV multispectral imagery for wheat trait prediction. BN denotes batch normalization.

Alongside conventional ML frameworks such as SVR and RFR, a classical DL method, the fully connected forward deep neural networks (DNN), was selected for wheat GY, GP, and TW prediction using the handcrafted features as inputs. DNN, also known as a multi-layer perceptron (MLP), consists of multiple interconnected layers of neurons that enable the model to learn complex patterns from large datasets. Each layer progressively extracts abstract representations from the input features and captures both linear and nonlinear relationships (Yi et al., 2016). DNN has also been widely applied in remote sensing tasks such as yield prediction (Anand and Jhajharia, 2025). In this study, multiple DNN architectures were evaluated through training and cross-validation, and the best-performing architecture was selected for implementation. The input feature dimension for DNN is 1D input vector with 440 features per sample, represented as (440,). The details of the selected DNN architecture are shown in **Figure 4**.

##### 3.2.1.4 One dimensional CNN

CNN is one of the most broadly used DL approaches. It is primarily designed to process spatially and sequentially structured data, making it highly effective for image-based analysis. Unlike fully connected DNN, CNN architectures leverage convolutional operations to autonomously derive hierarchical feature sets, enabling the simultaneous detection of intricate local details and overarching global spatial arrangements (Krizhevsky et al., 2012). 1D-CNN applies convolutional operations along the single input dimension, making it particularly useful for structured 1D numerical inputs, such as time series or handcrafted feature sets (Mao et al., 2023). 1D-CNN has been widely utilized in multitemporal remote sensing for processing time-series data (Gupta et al., 2024) and in hyperspectral image analysis for extracting spectral feature representations from 1D inputs (Saini et al., 2024). In this study, multiple 1D-CNN architectures were evaluated through training and cross-validation, and the best-performing architecture was selected for implementation. Unlike SVR, RFR, and DNN, 1D-CNN applies convolutional operations along a single dimension. Consequently, its input feature dimension is expressed as (440, 1), representing a 1D sequence of 440 features with a single channel per sample. The architectural specifications and layer configurations for the implemented 1D-CNN are detailed in **Figure 4**.

#### 3.2.2 End-to-end direct image-based modeling approach

In comparison with handcrafted feature-based modeling approach, an end-to-end imagery-based modeling method, which utilizes multispectral image patches from each plot as input, was also explored. Specifically, DL models capable of directly handling raw image inputs, including 2D-CNN, 3D-CNN, and a hybrid 2D-CNN with Long Short-Term Memory (LSTM), denoted as 2D-CNN-LSTM, were selected to predict GY, GP, and TW of wheat.

##### 3.2.2.1 Two dimensional CNN

2D-CNN is one of the most commonly used CNN architectures, it automatically extracts spatial features from input image patches through convolutional operations (Krizhevsky et al., 2012). It has been widely used for remote sensing applications to capture spatial patterns and complex relationships from input multispectral and hyperspectral imagery, especially in the case of high-resolution remote sensing imagery for land use/cover, crop species classification, change detection, as well as crop monitoring and yield prediction. In this study, image patches from each plot per flight were resized to 224 × 224 × 5 to match the input requirements of 2D-CNN models. For multitemporal analysis, images from five flights (growth stages) were stacked, resulting in an input dimension of 224 × 224 × 25 for 2D-CNN models. The details of the selected 2D-CNN architecture are shown in **Figure 4**.

##### 3.2.2.2 3D convolutional neural network

3D-CNN extends the traditional 2D-CNN by applying convolutions across an additional dimension, such as the spectral or temporal dimension of remote sensing imagery (Hamida et al., 2018). Unlike 2D-CNN, which processes images as independent 2D slices, 3D-CNN analyzes volumetric data, making it well-suited for multitemporal remote sensing and multispectral/hyperspectral imagery with rich spectral depth. This capability allows 3D-CNN to simultaneously preserve spatial, spectral, and temporal dependencies, enhancing feature representation and predictive accuracy in multitemporal multispectral remote sensing applications (El Sakka et al., 2025). In this study, multitemporal UAV image patches from five wheat growth stages were stacked into a 4D input tensor with a shape of (5 × 224 × 224 × 5), where the first dimension represents the number of time steps (flights), 224 × 224 denotes the spatial resolution, and the last dimension corresponds to the five multispectral bands. This configuration allows the 3D-CNN to jointly learn spatial, spectral, and temporal representations simultaneously. The details of the selected 3D-CNN architecture are provided in **Figure 4**.

##### 3.2.2.3 Hybrid 2DCNN and long short term memory

A hybrid 2D-CNN and Long Short Term Memory (LSTM), denoted as 2DCNN-LSTM, combines 2D-CNN for spatial feature extraction with LSTM to capture temporal dependencies in multitemporal imagery data (Tulapurkar et al., 2020). Specifically, it applies a 2D-CNN to extract spatial features from the UAV multispectral image patches from each day and then feeds those features as a sequence into an LSTM to model the temporal dynamics. Unlike 2D-CNN, which only analyzes spatial patterns, or 3D-CNN, which processes volumetric data but often struggles with long-term temporal relationships, 2DCNN-LSTM can often sequentially integrate spatial features over time, improving the representation of dynamic crop growth patterns (Rußwurm and Körner, 2018). This hybrid approach can often enhance predictive accuracy in multitemporal remote sensing applications (Cheng et al., 2023). Similar to 3D-CNN, the input dimension for 2DCNN-LSTM models is expressed as t × 224 × 224 × b. The details of the selected 2DCNN-LSTM architecture are presented in **Figure 4**.

### 3.3 Model training and evaluation

For both handcrafted feature-based and imagery-based models, the dataset was randomly partitioned into a training set and an independent test set using a 70/30 split. Model fitting was conducted using the 70% portion, and all performance reporting was based solely on predictions for the held-out 30% test data. Predictive accuracy was quantified with the root mean square error (RMSE), the relative RMSE (RMSE%), and the coefficient of determination (R²) (Equations 1–3). In these metrics, $y_{i}$ denotes the observed value and ${\hat{y}}_{i}$the corresponding model estimate for grain yield, grain protein, or test weight; $\bar{y}$ is the mean of the observed values, and n represents the number of samples in the test set.

(1) $$RMSE=\sqrt{\frac{\sum_{i=1}^{n}{\left(y_{i}-{\hat{y}}_{i}\right)}^{2}}{n-1}}$$

(2) $$RMSE\%=\frac{RMSE}{\bar{y}}*100$$

(3) $$R^{2}=1-\frac{\sum_{i=1}^{n}{\left(y_{i}-{\hat{y}}_{i}\right)}^{2}}{\sum_{i=1}^{n}{\left(y_{i}-\bar{y}\right)}^{2}}$$

All DL models were developed in the Keras API running on TensorFlow. Hidden layers, including both convolutional blocks and dense layers, used the ReLU nonlinearity, whereas the output layer employed a linear activation to produce continuous predictions (**Figure 4**). Models were trained by minimizing mean squared error (MSE), with parameter updates performed using the Adam optimization algorithm. Key training settings (e.g., learning rate, dropout probability, and mini-batch size) were optimized for each architecture (**Table 1**). Based on preliminary trials comparing 50, 100, and 150 epochs, we selected 50 training epochs, and a batch size of 32 was adopted after testing candidate sizes of 16, 24, and 32.

**Table 1:** Parameter settings for the traditional ML and DL models that were used to predict the GY, GP, and TW.

Model	Parameters	Parameter tuning
Support Vector Regression (SVR)	kernel	rbf, poly, linear
	C	0.01,0.125, 0.25, 0.5, 1.0, 2.0, 4.0
	gamma	1, 0.1, 0.01, 0.001, 0.0001, 0.0005
Random Forest (RFR)	n_estimators	50, 100,….,1000 (interval 50)
	max_features	auto, log2, sqrt,
	criterion	absolute_error, poisson, squared_error
Deep Neural Networks (DNN), 1D/2D/3D Convolutional Neural Networks (CNN), 2DCNN-Long Short Term Memory Network (LSTM)	Optimizer	Adam
	Batch size	8,16,32
	Number of epochs	50, 100
	Activation function	ReLu, Linear
	Learning rate	0.1, 0.01, 0.001, 0.0001, 0.00001
	Dropout	0.1, 0.2, 0.3, 0.4, 0.5

To reduce the risk of overfitting, cross-validation was applied during model training and hyperparameter tuning using the training dataset. Model performance was then evaluated on an independent held-out test set. In addition, the dropout layer was incorporated into the deep learning architectures, as it is a widely used regularization technique to reduce overfitting in deep learning models (Salehin and Kang, 2023). Batch normalization was also used to improve training stability and support better generalization, as it can enhance optimization stability and may also provide a regularizing effect during training (Garbin et al., 2020).

**Figure 5:**
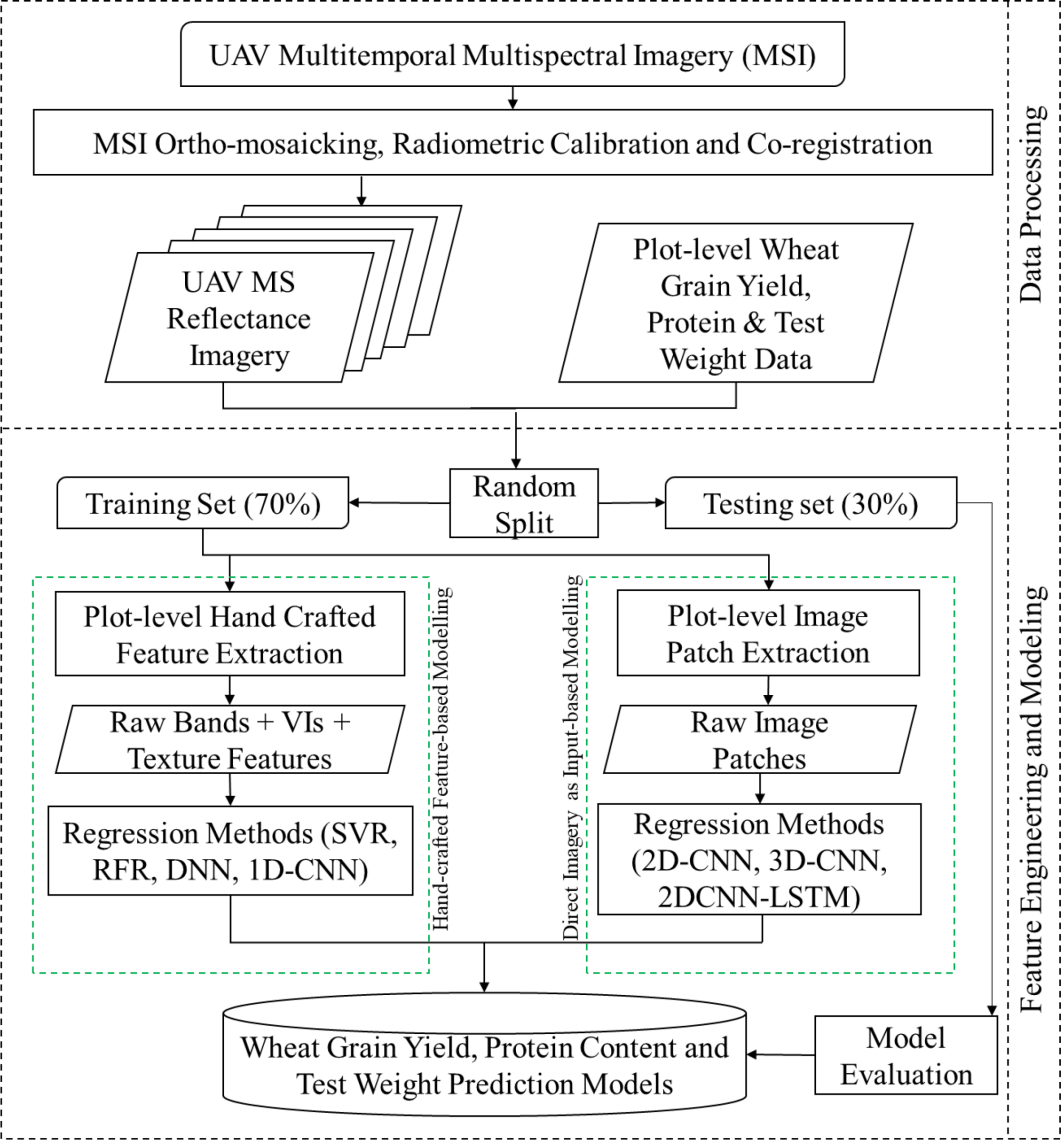
Workflow diagram of UAV-based multispectral imagery processing and modeling for wheat trait prediction. Key steps include image acquisition, preprocessing, feature extraction (e.g., vegetation indices), and model development using various machine learning and deep learning approaches. VIs, vegetation indices; SVR, support vector regression; RFR, random forest regression; DNN, deep neural network; CNN, convolutional neural network; 1D, one-dimensional; LSTM, long short-term memory.

The overall workflow of the methods, including feature extraction, model training, and assessment procedures, is summarized in **Figure 5**.

## 4 Results

### 4.1 Wheat grain yield prediction analysis

Wheat GY prediction was performed using both handcrafted feature-based (using UAV multitemporal multispectral image-derived spectral and texture features as inputs) and imagery-based (using multitemporal multispectral raw imagery as input) approaches across a range of ML and DL models. As shown in the model testing results in **Table 2**, among the handcrafted feature-based models, DNN achieved the best performance with an R² of 0.63 and the lowest RMSE% of 10.09%. SVR and RFR followed closely, both with an R² of 0.63 and 0.62 and RMSE% values of 10.14% and 10.17%, respectively. The 1D-CNN model showed the weakest performance within this group, with the lowest R² (0.61) and the highest RMSE% (10.64%). In comparison, the imagery-based models generally slightly outperformed the handcrafted feature-based models. Specifically, the 3D-CNN achieved the highest overall performance, with an R² of 0.65 and the lowest RMSE% of 10.00%. The 2D-CNN model performed similarly, with an R² of 0.64 and an RMSE% of 10.19%. The 2DCNN-LSTM model showed reduced performance, with a lower R² of 0.61 and a higher RMSE% of 10.50%. Overall, imagery-based models, particularly the 3D-CNN model demonstrated slightly superior predictive performance for wheat GY estimation in terms of both R² and RMSE%, compared to handcrafted feature-based approaches.

**Table 2:** Model testing results for predicting wheat grain yield (GY, kg/ha), grain protein content (GP, %), and grain test weight (TW, g/l) using handcrafted feature–based and imagery-based approaches across the different models.

		Handcrafted feature-based approach				Imagery-based approach		
Target	Metrics	SVR	RFR	DNN	1D-CNN	2D-CNN	3D-CNN	2DCNN-LSTM
Grain Yield (GY)	R^2^	0.63	0.62	0.63	0.61	0.64	0.65	0.61
	RMSE	458.3	459.6	456.0	480.9	460.5	452.0	474.6
	RMSE%	10.14%	10.17%	10.09%	10.64%	10.19%	10.00%	10.5%
Grain Protein Content (GP)	R^2^	0.61	0.60	0.40	0.50	0.61	0.61	0.48
	RMSE	0.76	0.80	0.95	0.86	0.76	0.76	0.88
	RMSE%	5.54%	5.86%	6.91%	6.31%	5.54%	5.54%	6.40%
Grain Test Weight (TW)	R^2^	0.66	0.65	0.67	0.61	0.68	0.69	0.64
	RMSE	21.7	22.7	21.4	24.6	20.7	20.5	23.3
	RMSE%	2.71%	2.89%	2.66%	3.23%	2.53%	2.49%	3.00%

### 4.2 Wheat grain protein content prediction analysis

Similar to GY prediction, GP was also performed using both handcrafted feature-based (using UAV multitemporal multispectral image-derived spectral and texture features as inputs) and imagery-based (using multitemporal multispectral raw imagery as input) approaches across a range of ML and DL models. Based on the model testing results outlined in **Table 2**, among the handcrafted feature-based models, SVR achieved the best performance with an RMSE% of 5.54% and an R² of 0.61, followed closely by RFR (RMSE% = 5.86%, R² = 0.60). DNN showed the weakest performance in this group, with the highest RMSE% (6.91%) and the lowest R² (0.40), while 1D-CNN performed moderately with an RMSE% of 6.31% and an R² of 0.50. In terms of the imagery-based models, both 2D-CNN and 3D-CNN achieved the best results, each with an RMSE% of 5.54% and an R² of 0.61. Meanwhile, 2DCNN-LSTM showed reduced performance, with a lower R² of 0.48 and a higher RMSE% of 6.40%. Overall, the imagery-based models, particularly the 2D-CNN and 3D-CNN, performed comparably to the best-performing handcrafted feature-based models in predicting wheat GP, based on both R² and RMSE%.

### 4.3 Wheat grain test weight prediction analysis

Following the same modeling strategy applied to wheat GY and GP, TW was also predicted using both handcrafted feature-based (using UAV multitemporal multispectral image-derived spectral and texture features as inputs) and imagery-based (using multitemporal multispectral raw imagery as input) approaches across a range of ML and DL models. As shown in the model testing results presented in **Table 2**, the best performance from the handcrafted feature-based approach was achieved by DNN with an RMSE% of 2.66 and an R² of 0.67. SVR followed with an RMSE% of 2.71 and an R² of 0.66, while RFR resulted in an RMSE% of 2.89 and an R² of 0.65. The weakest performance was observed with 1D-CNN, which yielded an RMSE% of 3.23 and an R² of 0.61. For the imagery-based approach, 3D-CNN achieved the highest overall performance with an RMSE% of 2.49 and an R² of 0.69. The 2D-CNN also performed well with an RMSE% of 2.53 and an R² of 0.68. In comparison, 2DCNN-LSTM showed lower performance, with an RMSE% of 3.00 and an R² of 0.64, indicating limited benefit from the temporal modeling component for TW prediction. Overall, the results show that the imagery-based models, especially 3D-CNN and 2D-CNN, outperformed all models used in the handcrafted feature-based approach.

### 4.4 Optimal UAV imaging time for wheat GY, GP, and TW prediction

**Figure 6:**
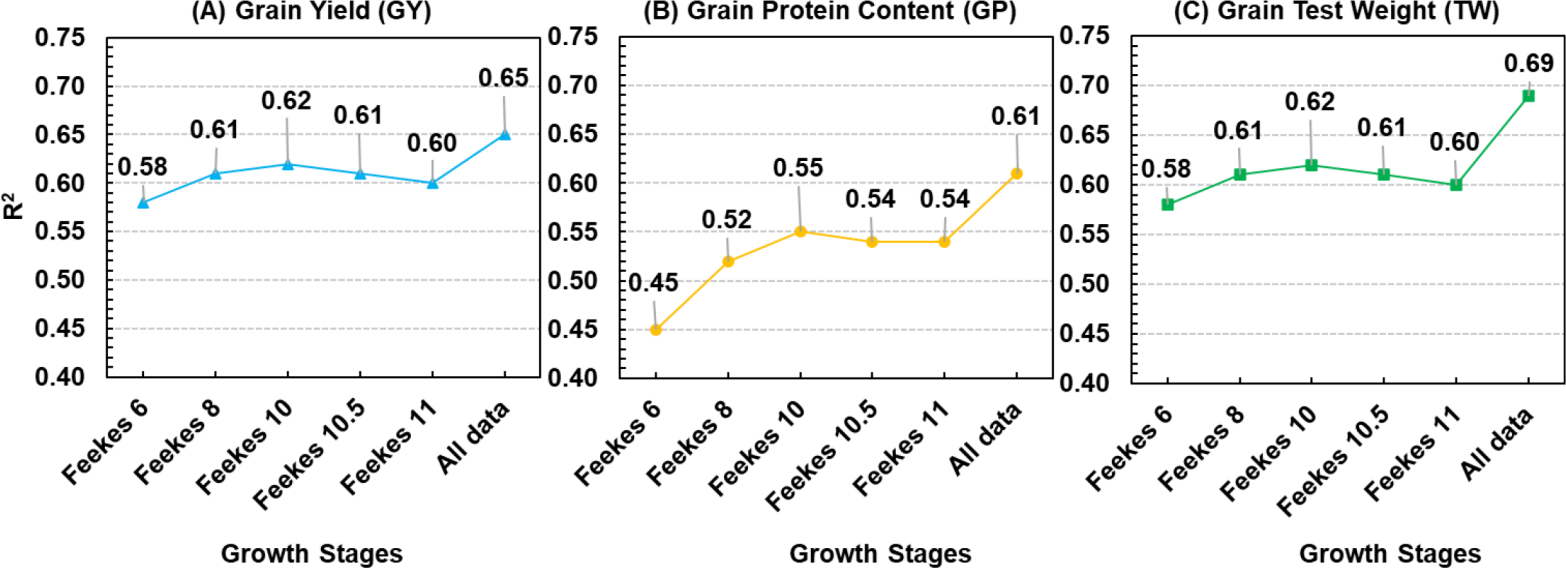
The 3D-CNN model testing performance (R²) for wheat grain yield (GY) **(A)**, grain protein content (GP) **(B)**, and grain test weight (TW) **(C)** based on UAV multispectral imagery collected at five key growth stages (Feekes 6, 8, 10, 10.5, and 11), as well as using combined data from all stages.

Since the 3D-CNN model demonstrated relatively stable and strong performance across wheat GY, GP, and TW prediction tasks, it was selected to evaluate the predictive capability of UAV multispectral imagery collected at different wheat growth stages (i.e., Feekes 6, 8, 10, 10.5, and 11). The objective was to identify the optimal imaging time for each trait. This evaluation was performed separately for GY, GP, and TW. As shown in **Figure 6**, for GY prediction, model testing R² values increased from 0.58 at Feekes 6 to 0.62 at Feekes 10, followed by a slight decline to 0.60 at Feekes 11. Feekes 10 achieved the highest R² among all single-stage UAV data. When UAV imagery from all growth stages was combined, the 3D-CNN model achieved an R² of 0.65, outperforming any single-stage data-based prediction.

For wheat GP prediction using single-stage UAV data, the model testing R² increased from 0.45 at Feekes 6 to a peak of 0.55 at Feekes 10, then remained consistent at 0.54 through Feekes 10.5 and 11. When UAV data from all growth stages were combined, the model achieved a higher R² of 0.61, outperforming any single-stage input. For TW prediction, the model testing R² rose from 0.58 at Feekes 6 to a maximum of 0.62 at Feekes 10, followed by a slight decline to 0.60 at Feekes 11. When imagery from all stages was used, the R² further improved to 0.69.

## 5 Discussion

### 5.1 Comparison of handcrafted feature-based and imagery-based modeling approaches

**Figure 7:**
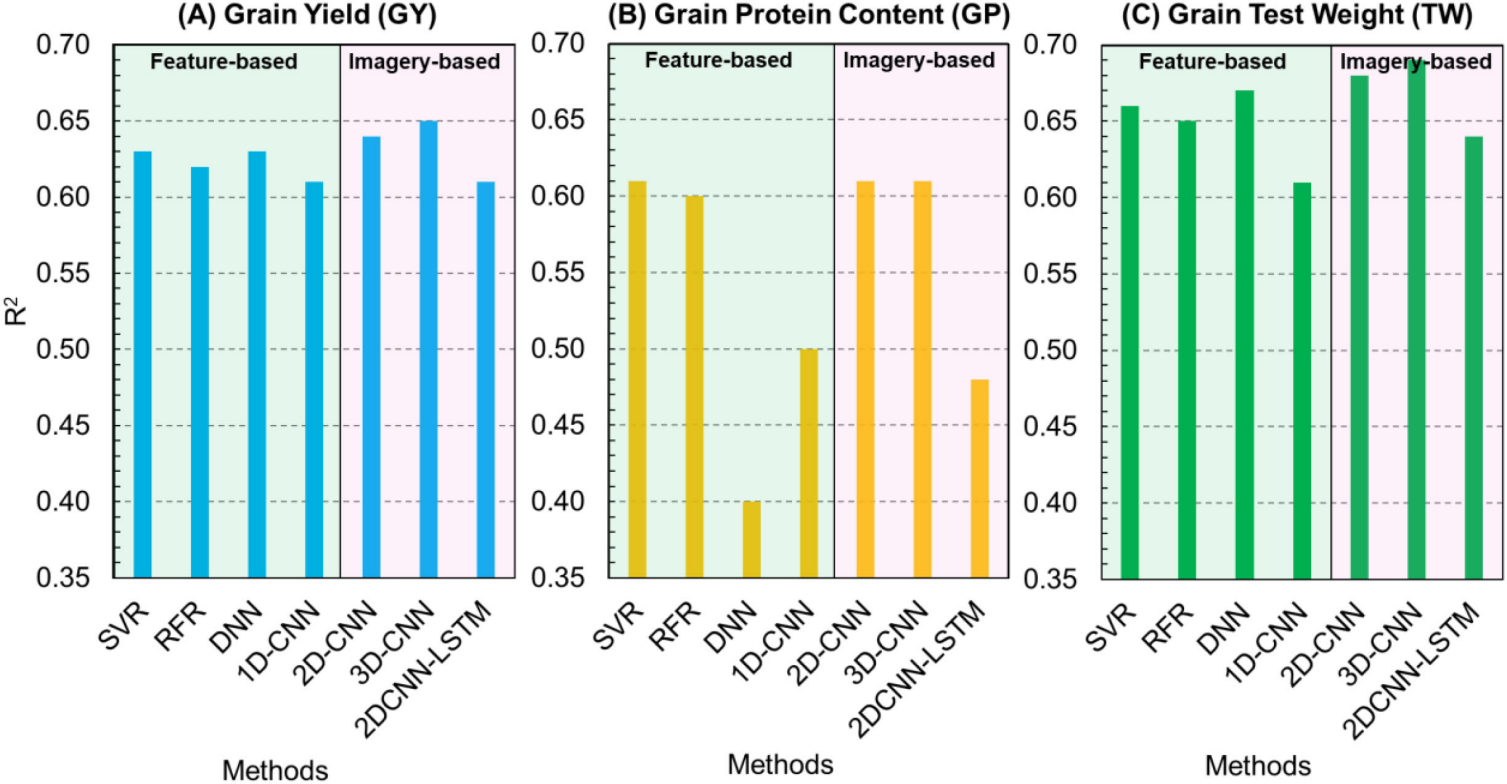
Comparison of model testing performance (R²) for wheat grain yield [left **(A)**, blue], grain protein content [center **(B)**, yellow], and grain test weight [right **(C)**, green] using different modeling approaches. Models are categorized into feature-based (SVR, RFR, DNN, 1D-CNN) and direct imagery-based methods (2D-CNN, 3D-CNN, 2DCNN-LSTM).

**Figure 7** provides a comparative overview of prediction performance between handcrafted feature-based and imagery-based approaches. Across all three wheat traits, including GY, GP, and TW, imagery-based models delivered comparable or slightly superior accuracy. This trend was consistently observed for each wheat trait. The improved performance is likely due to the ability of the imagery-based approach to directly learn complex, hierarchical features, including but not limited to spatial, spectral, and textural features from UAV imagery, without being constrained by the limitations of manually engineered features (Joshi et al., 2023). This end-to-end learning capability helps preserve fine-scale variability and subtle spectral patterns that are often lost during traditional feature extraction (Reddy et al., 2024). The effectiveness of the direct imagery-based approach demonstrated in this study aligns with findings from previous research (Bhadra et al., 2024; Sagan et al., 2021).

The feature-based approach is widely adopted in UAV remote sensing-based HTP communities, in part due to its implementation simplicity and ease of interpretability (Dhakal et al., 2023; Tanaka et al., 2024). However, in this study, it yielded comparable but slightly poorer performance compared to the imagery-based approach. This performance gap may be attributed to several factors. First, the plot-level averaging of UAV imagery can potentially lead to the loss of fine-grained spatial information inherent in high-resolution data, which may limit the model’s ability to capture subtle spatial variability. Additionally, relying on a limited set of predefined handcrafted features may overlook critical patterns present in the imagery, and potentially reduce the overall predictive capacity of the model (Shrestha et al., 2024).

The imagery-based approach streamlines the modeling process by eliminating manual feature engineering and allowing models to automatically derive meaningful representations from the raw data. However, it is important to note that DL models that are often utilized for imagery-based approaches, such as 2D-CNN, 3D-CNN, 2DCNN-LSTM, and Vision Transformers (ViT), typically require larger training datasets, longer training times, and increased computational resources (Boletsis et al., 2023). Therefore, these approaches should be considered not only in terms of predictive performance, but also with respect to efficiency, scalability, and generalizability across different crop types, environments, and target traits.

Regarding the comparison between handcrafted feature-based and imagery-based modeling workflows and input representations, the handcrafted feature-based approach typically extracts features from imagery and summarizes them as average pixel values, often at the field-plot or sampling-spot level (Sagan et al., 2021). This has been the most commonly adopted workflow in previous studies (Kaushal et al., 2024; Maimaitijiang et al., 2025), likely due to its effectiveness in deriving informative features and achieving strong predictive performance.In contrast, imagery-based deep learning is specifically designed to learn directly from spatially organized image inputs (Wang et al., 2022). A more controlled comparison using a similar input structure for both workflows would require converting imagery into a dimension-reduction representation for the feature-based approach, for example, through flattening or dimensionality reduction methods (Gadekallu et al., 2021; Sugumar and Suganya, 2023). However, such a transformation converts chip- or patch-level imagery from each plot into a one-dimensional pixel vector, which may reduce or eliminate the spatial information that imagery-based deep learning models are specifically designed to exploit (Zhao and Du, 2016). For this reason, the present study compared the two workflows in forms that are more representative of their common use in the literature, rather than as a fully controlled comparison with identical input representations. Although imagery-based deep learning may preserve richer spatial information and reduce the need for manual feature engineering (Wang et al., 2022), these potential advantages should be considered alongside practical limitations, including more complex data preparation, longer training time, and greater computational cost. Therefore, the choice between feature-based and imagery-based workflows should depend not only on predictive performance but also on the intended application, available computational resources, and the importance of model interpretability.

### 5.2 Performance of machine and deep learning models

As shown in **Figure 7**, overall, across all three wheat traits, DL-based models, particularly 2D-CNN and 3D-CNN, consistently outperformed traditional ML models such as SVR and RFR, as well as the DNN and 1D-CNN models. The performance advantage of the 3D-CNN is plausibly explained by its capability to extract joint spatiotemporal-spectral features from multitemporal and multispectral imagery (Ji et al., 2018), which has proven effective for capturing complex agronomic signals related to biomass accumulation and yield formation (Nevavuori et al., 2020). Moreover, 3D-CNN preserves spatial-temporal context throughout the convolutional process, offering a distinct advantage under sparse or irregular temporal sampling conditions, as was the case in this study (Nevavuori et al., 2020). These findings are consistent with previous studies demonstrating the robustness of 3D-CNN in remote sensing-based plant trait prediction tasks (Bhadra et al., 2024; El Sakka et al., 2025). Although 2D-CNN primarily captures spatial features, it still delivers slightly lower yet competitive performance, underscoring the importance of high-resolution spatial information from UAV imagery in the accurate prediction of wheat yield and grain quality traits.

While the 2DCNN-LSTM architecture is designed to capture spatial-spectral patterns at each time step and can model temporal dependencies across multitemporal multispectral remote sensing imagery, it demonstrated comparatively weaker performance than 3D-CNN and other models across all wheat trait predictions. One possible explanation is that CNN-LSTM models typically require a sufficient number of temporally rich observations to effectively learn meaningful growth dynamics and phenological trends. LSTM-based models are intended to learn temporal progression and sequential dependencies; therefore, their effectiveness generally depends on a sufficiently dense, temporally regular sequence of observations (Song et al., 2025). In this study, the UAV data were collected on only five separate days during the growing season: May 18 (Feekes 6), June 3 (Feekes 8), June 9 (Feekes 10), June 27 (Feekes 10.5), and July 7 (Feekes 11). These acquisitions were separated by uneven intervals of 16, 6, 18, and 10 days, respectively, and provided limited temporal depth, which likely limited the model’s ability to capture continuous temporal progression. Consequently, the limited number of acquisitions and the irregular spacing between flight dates in this study may have led the LSTM component to provide little meaningful temporal information beyond what was already captured by the convolutional layers, and likely constrained the CNN-LSTM model’s ability to learn continuous crop developmental patterns and phenological transitions. This observation aligns with previous findings that emphasize the importance of dense and continuous time-series inputs for the success of CNN-LSTM-based models in remote sensing applications (Fu et al., 2024). Furthermore, the LSTM component in the 2DCNN-LSTM architecture introduces additional trainable parameters, increasing model complexity and training demands, which can be particularly challenging in scenarios with limited training samples (Koşar and Barshan, 2023). This result suggests that CNN-LSTM architectures are likely to be more effective when trained on more densely sampled UAV time series, which better exploit their temporal modeling capabilities.

Another important observation from the analysis was that the DNN model achieved the best performance within the feature-based workflow for wheat TW prediction. A plausible explanation is that TW was likely associated with the combined effects of complex, nonlinear combinations of multiple UAV-derived spectral and texture predictors, which represented heterogeneous plot-level information (Kaushal et al., 2024). Therefore, the fully connected DNN architecture for TW prediction is likely better suited to learning global nonlinear relationships across the full feature set used in this study (Habeeb and Mustafa, 2024). Additionally, wheat TW likely showed better predictive performance and lower RMSE% than GP or GY, because it reflects the physical grain quality (Vyn and Tollenaar, 1998) outcome linked to grain filling and kernel condition (Nuttall et al., 2017), which can be indirectly captured by UAV-derived canopy spectral and texture features. In comparison, wheat GP depends more strongly on biochemical nitrogen dynamics (Martre et al., 2006), which may be less directly observable from multispectral canopy imagery, and GY integrates a broader set of seasonal processes and yield components.

### 5.3 Predictivity of UAV multispectral data of wheat GY, GP, and TW

**Figure 8:**
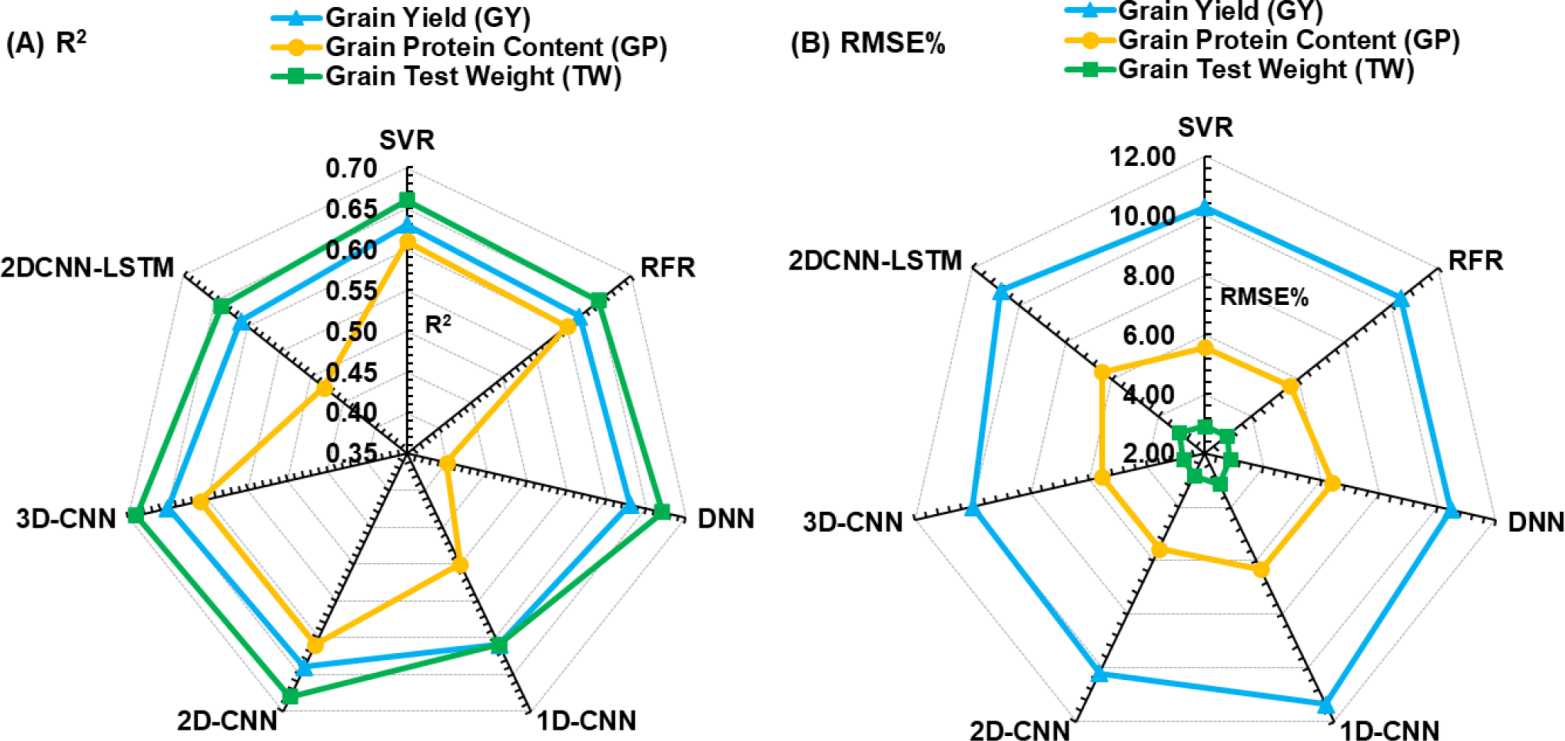
Comparison of model testing performance [R² **(A)** and RMSE% **(B)** values] for predicting wheat GY, GP, and TW when using SVR, RFR, DNN, 1D-CNN, 2D-CNN, 3D-CNN, and 2DCNN-LSTM models.

UAV multispectral imagery demonstrated varying levels of predictive performance across wheat GY, GP, and TW as evaluated using R², RMSE, and RMSE% (**Figure 8**; **Table 2**). GY and TW exhibited higher R² values than GP, and TW showed the lowest RMSE% across models (2.49%–3.23%) together with relatively high R² values (0.61–0.69). Notably, predictions for GY and TW were relatively strong and consistent across models in this work. GY also showed relatively strong performance, with R² values ranging from 0.61 to 0.65. However, RMSE% remained around 10.00%–10.64%, reflecting the broader numerical range and greater overall variability of GY. By comparison, GP showed lower and more variable predictive performance, with R² values ranging from 0.40 to 0.61 and RMSE% values of 5.54%–6.91%. The relatively strong overall performance of wheat GY and TW is closely linked to kernel development, starch accumulation, and grain filling efficiency (Kumar et al., 2017). These physiological processes are strongly influenced by canopy structure, biomass accumulation, and plant vigor, which are often well captured by UAV-based high-resolution imagery through features such as canopy density, height variability, vegetation indices, and texture metrics, potentially leading to better predictions (Dilmurat et al., 2022; Kaushal et al., 2024).

Regarding wheat TW prediction, TW was considered one of the important primary target traits in this study rather than a secondary validation variable. As a grain-quality trait, TW adds a physical quality dimension to UAV-based wheat trait estimation, complementing GY and GP prediction. While GP is more closely related to grain composition, TW reflects grain physical quality and therefore broadens the assessment of wheat grain quality prediction performance. Its consistent predictability and low RMSE% in this study indicate that multispectral and texture information contained meaningful trait-related information linked to final grain physical quality. The broadly similar performance pattern observed for TW suggests that the UAV imagery may have captured related canopy-level signals associated with crop growth, biomass status, grain filling, and grain development (Kumar et al., 2017; Dilmurat et al., 2022; Kaushal et al., 2024). This is particularly noteworthy because TW appears to be less frequently investigated than GY and GP in UAV-based wheat prediction studies, suggesting that its successful in-season prediction represents a useful extension of current UAV-based HTP applications.

**Figure 9:**
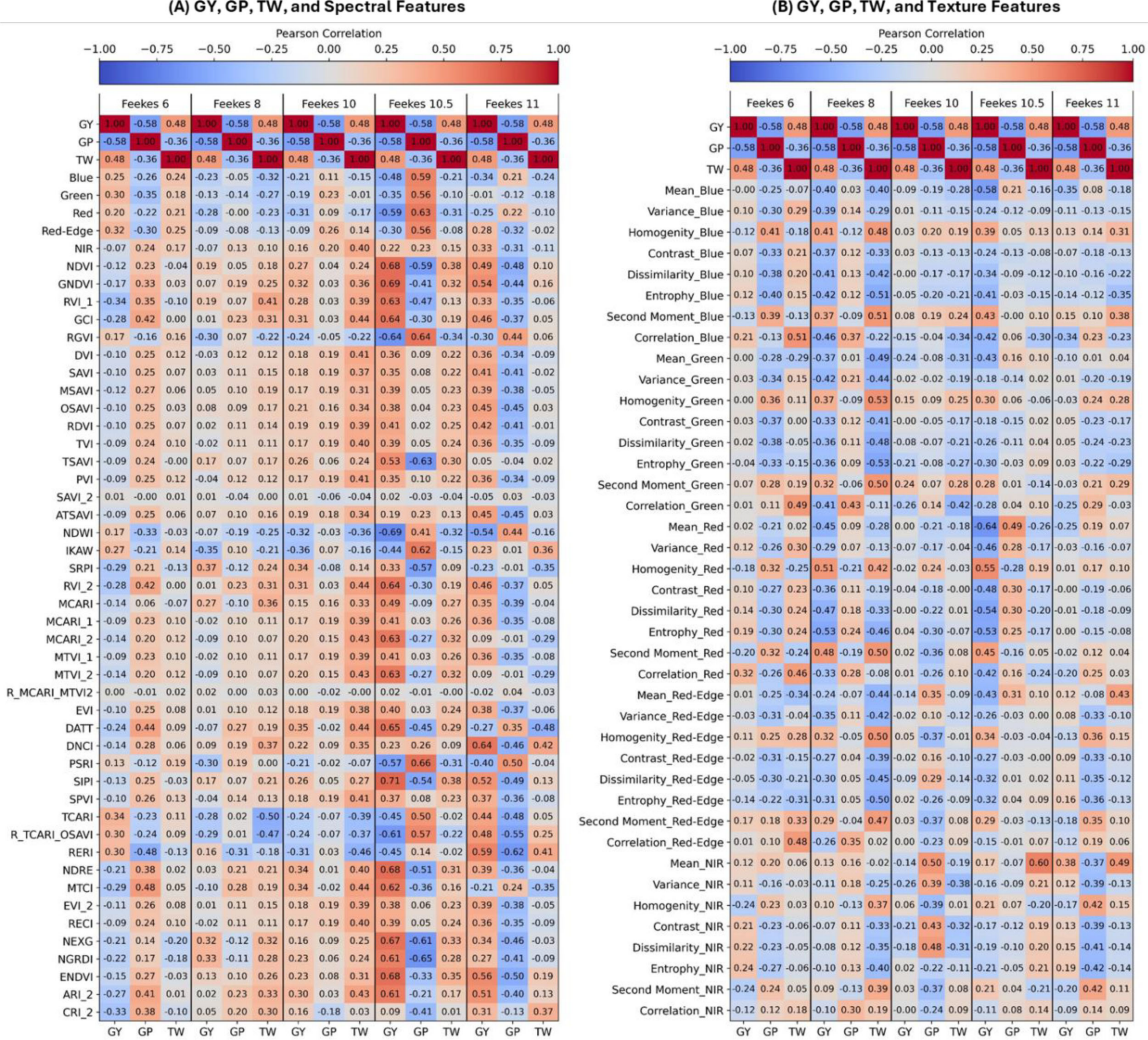
Pearson correlation heatmaps showing relationships among wheat grain yield (GY), grain protein content (GP), grain test weight (TW), and UAV-derived features across Feekes 6, Feekes 8, Feekes 10, Feekes 10.5, and Feekes 11. **(A)** Correlations between wheat GY, GP, TW and spectral features. **(B)** Correlations between wheat GY, GP, TW and GLCM texture features derived from UAV multispectral image bands. Positive correlations are shown in red and negative correlations in blue.

The correlation analysis among wheat traits and UAV-imagery derived features (**Figures 9A, B**) further indicated that TW was not simply a proxy trait for GY, although GY and TW were moderately correlated (Pearson’s r = 0.48; **Figure 9**). In addition, several UAV-imagery-derived spectral and GLCM texture features showed moderate correlations with TW, and some of these associations were comparable to or stronger than those with GY. This pattern was particularly evident for spectral features, such as NIR, from Feekes 10 (at optimal UAV imaging time), and for texture features, such as homogeneity, which may reflect within-plot spatial uniformity. Additionally, a more uniform and physiologically active canopy during reproductive and grain-filling stages may contribute to more stable assimilate production and translocation to developing kernels, whereas spatially heterogeneous canopy conditions may reflect stress, uneven growth, lodging, canopy gaps, or variable senescence, which can affect grain filling and final grain density. Thus, the strong TW prediction performance and low RMSE% likely serve as evidence that UAV-imagery-derived spectral and texture information contained meaningful indirect signals related to final grain physical quality, rather than as evidence that TW was predicted only through its correlation with GY.

In terms of wheat GP prediction, the performance showed less accurate and more variable across models, compared to the prediction of wheat GY and TW. This is likely because protein content is primarily governed by internal nitrogen metabolism and complex biochemical pathways (Abiola et al., 2024) that are not directly observable through canopy-level spectral or spatial features captured by UAV imagery. Additionally, the limited spectral resolution of multispectral sensors further constrains their ability to detect subtle biochemical signals related to nitrogen dynamics and protein accumulation (Ma et al., 2022). As a result, UAV multispectral imagery remains less effective for estimating biochemically complex traits such as GP. These limitations indicate that improving GP prediction may consider integrating multimodal UAV data by combining hyperspectral imaging and LiDAR sensing, which are capable of capturing subtle biochemical and biophysical signals more effectively (Li et al., 2022). In addition, exploring advanced DL techniques for fusing multimodal data sources could further enhance the predictive accuracy of wheat trait estimation.

### 5.4 Influence of UAV imaging time on wheat GY, GP, and TW prediction

The timing of UAV imagery acquisition often impacts the accuracy of wheat trait prediction models. Previous studies have shown that multitemporal UAV flights can often lead to improved wheat grain yield predictions (Camenzind and Yu, 2024). However, operational constraints such as flight costs, weather limitations, and potential technical issues make frequent UAV missions challenging in real-world scenarios (Makam et al., 2024). In this study, UAV multispectral imagery was collected at five critical wheat growth stages: Feekes 6 (jointing), Feekes 8 (flag leaf), Feekes 10 (booting), Feekes 10.5 (heading), and Feekes 11 (grain filling). It is important to note that, due to growth variation among different wheat genotypes, the timing of growth stages may have varied by up to ±3 days at the time UAV flights were conducted in this study. As shown in **Figure 6**, combining UAV data from multiple growth stages resulted in improved prediction performance compared to using data from any single stage alone, which is consistent with findings from several previous studies (Camenzind and Yu, 2024; Nevavuori et al., 2020). Among the individual growth stages, imagery acquired at the Feekes 10 stage (booting to heading) yielded slightly better performance for all three traits, GY, GP, and TW, compared to other stages evaluated in this study. However, it should be noted that this finding and the identification of Feekes 10 as the best single-stage timing in this study should be considered in the context of the 3D-CNN model, which showed the best and most consistent performance across all three traits. Therefore, this result is likely model-dependent rather than fully model-independent. Other feature-based and imagery-based models may also be examined for identifying optimal imaging time, and, if consistent across all three traits, may be used in broader evaluation, although their overall model performance is not consistent across traits. Moreover, this observation and optimal imaging time broadly align with previous findings indicating that wheat yield predictions tend to be most accurate when UAV data are collected during the Feekes 10.0–10.5 growth stages (Zhou et al., 2023). It is also worth noting that some studies have identified the flowering stage as the most effective timing for yield prediction (Yang et al., 2025).

For wheat GY and TW, which are often strongly influenced by canopy architecture and biomass accumulation prior to grain filling, the spectral and structural signals captured at Feekes 10 effectively reflect plant vigor and pre-grain development capacity (Walsh et al., 2023). This may explain the slightly better prediction performance observed with UAV imagery collected at this stage (Yu, 2025). In the case of wheat GP, which is generally more challenging to predict due to the complex influence of nitrogen metabolism, UAV data from Feekes 10 still yielded marginally better predictions. This is likely because canopy-level spectral features at this stage, such as red-edge and near-infrared (NIR) reflectance, may indirectly capture signals related to nitrogen uptake and redistribution potential more effectively than data from other stages (Walsh et al., 2022). Future work could explore more frequent UAV flights (e.g., weekly or sub-weekly) over wheat fields under diverse environmental conditions to further evaluate which specific growth stages and timing consistently yield the most accurate trait predictions.

### 5.5 Study limitations and future research

This study has several limitations that should be acknowledged. First, the analysis was conducted using data from a single growing season, which may limit the generalizability and robustness of the models across multiple growing seasons. Second, the study relied on multispectral imagery, which may have limited capacity to capture the detailed spectral information needed for predicting biochemically complex traits, particularly grain protein. Future studies may therefore consider integrating multimodal UAV data, such as hyperspectral and LiDAR observations, to better capture biochemical and structural trait-related signals. Third, the comparison between handcrafted feature-based and imagery-based workflows should be interpreted as a comparison between two complete workflows rather than a fully controlled comparison, because the two approaches differ in both input representation and model architecture. Although, imagery-based methods may preserve richer spatial information, they also require more complex data preparation and greater computational cost. Fourth, the optimal imaging time identified in this study was based on the best-performing model. A more comprehensive evaluation using different models to determine the optimal imaging stage for each trait would provide stronger support for practical recommendations in high-throughput phenotyping studies. Fifth, the CNN-LSTM model may not have been fully utilized because only a limited number of multitemporal UAV acquisitions were available. Future studies should incorporate more continuous and denser temporal observations, which may improve model performance. Another limitation is that the dataset was randomly split into a training and a test set. Future studies may evaluate model performance across years and locations to better assess model generalizability. In addition, techniques such as transfer learning and multitask learning may further improve prediction performance for wheat traits. Future research should also explore more advanced techniques, such as attention-based deep learning and transformer-based architectures, which have shown strong potential in remote sensing and agricultural imaging tasks. Finally, implementing data augmentation strategies to expand the training dataset may improve both imagery-based and feature-based approaches by enhancing model robustness, reducing overfitting, and supporting better generalization across varying environments and genotypes.

## 6 Conclusion

This work highlights the value of integrating multi-date UAV multispectral observations with deep learning models to estimate important wheat traits such as GY, GP, and TW in field-scale high-throughput phenotyping. Two modeling strategies were evaluated: a conventional handcrafted feature-based approach using plot-level mean spectral and texture features as model input, and a direct image-based DL approach using raw UAV imagery as model input. In this study, the image-based modeling workflow showed comparable to modestly better performance than the handcrafted feature-based workflow across GY, GP, and TW predictions, with the 3D-CNN achieving the best overall results among the evaluated models. The 3D-CNN model can often capture complex spatial, spectral, and temporal relationships within the imagery, which potentially contributed to the highest overall accuracy for GY, GP, and TW predictions, with R² values of 0.65, 0.61, and 0.69, respectively. Importantly, the relatively strong and stable prediction of TW indicates that UAV multispectral imagery can support in-season prediction of grain physical quality in addition to GY and GP. These findings underscore the advantage of leveraging raw UAV imagery through end-to-end DL pipelines to preserve complex agronomic signals often lost in conventional handcrafted feature-based workflows.

This work also explored the impact of UAV image acquisition timing on model performance. The results revealed that within the 3D-CNN model-based analysis, imagery from the Feekes 10 stage (booting to heading) consistently produced better prediction accuracy than other individual stages. Feekes 10 often coincides with peak canopy development, biomass accumulation, and leaf area expansion, which are closely linked to yield formation and partially reflective of nitrogen dynamics, which may explain the improved predictions for both yield and protein traits using red-edge and NIR signals. Combining imagery from multiple stages further enhanced model performance across all traits, highlighting the value of multitemporal data for capturing wheat development dynamics.

Overall, this research underscores the promise of integrating UAV multispectral imaging with data-driven modeling to support high-throughput phenotyping and precision agriculture. The comparison between feature-based and image-based modeling workflows provides practical guidance for future implementation. While multitemporal imagery offers better prediction performance in this study, real-world constraints such as cost and weather may limit data collection frequency. Thus, identifying key phenological windows like Feekes 10 can provide an effective compromise. Future work should explore scalable DL architectures, including attention-based DL methods and Transformers, and evaluate these approaches across diverse growing conditions to improve model robustness and generalizability.

## Data Availability

The datasets presented in this article will be available upon request. Requests to access the datasets should be directed to Maitiniyazi Maimaitijiang, maitiniyazi.maimaitijiang@sdstate.edu and Sunish.Sehgal@sdstate.edu.
